# Long-term results of laparoscopic versus open intraperitoneal onlay mesh incisional hernia repair: a propensity score-matched analysis

**DOI:** 10.1007/s00464-018-6298-6

**Published:** 2018-06-25

**Authors:** Joël L. Lavanchy, Stefan E. Buff, Andreas Kohler, Daniel Candinas, Guido Beldi

**Affiliations:** Department of Visceral Surgery and Medicine, Inselspital, Bern University Hospital, University of Bern, 3010 Bern, Switzerland

**Keywords:** Incisional hernia repair, Long-term follow-up, Recurrence rates, Laparoscopic versus open operation technique

## Abstract

**Background:**

Intraperitoneal onlay mesh repair (IPOM) of incisional hernia is performed by laparoscopic and open access. The aim of the present study is to compare open versus laparoscopic surgery specifically using an IPOM technique for incisional hernia repair.

**Methods:**

A propensity score-matched observational single center study of patients that underwent IPOM between 2004 and 2015 was conducted. The primary outcome was hernia recurrence; secondary outcomes include length of stay, surgical site infections (SSI), complications, and localization of recurrence.

**Results:**

Among 553 patients with incisional hernia repair, 59% underwent laparoscopic and 41% open IPOM. A total of 184 patients completed follow-up. After a mean follow-up of 5.5 years recurrence rate was 20% in laparoscopic and 19% in open repair (*p* = 1.000). Patients undergoing laparoscopic IPOM had significantly reduced operation time (median 120 vs. 180 min, *p* < 0.001), shorter hospital stays (6 vs. 8 days, *p* = 0.002), less complications (10 vs. 23%, *p* = 0.046), and fewer SSI (1 vs. 21%, *p* < 0.001).

**Conclusions:**

Laparoscopic IPOM is associated with reduced morbidity compared to open IPOM for incisional hernia repair.

Incisional hernia is a frequent clinical challenge with an incidence between 11 and 23% after open abdominal surgery and is associated with relevant morbidity [1–4]. Trials and systematic reviews comparing laparoscopic with open surgical techniques showed reduced complications [5–7], less surgical site infections (SSI) [8–10], and a shorter hospital stay [5, 8, 9, 11] in patients undergoing laparoscopic incisional hernia repair. These studies, however, are confounded by the fact that not just the route of access but also the type of surgery is different. In open incisional hernia surgery, meshes are most frequently positioned in sublay or preperitoneal position, while in laparoscopic hernia surgery the most frequent operation is an intraperitoneal onlay mesh (IPOM) [12–14]. To avoid mesh position as confounding factor, the present study focuses solely on hernias repaired by an IPOM technique, by either laparoscopic or open access.

The objective of the present study is to describe long-term results of hernia recurrence after laparoscopic versus open IPOM incisional hernia repair and to compare anatomical details of hernia recurrence between these two techniques.

## Materials and methods

This retrospective cohort study is reported in accordance with the STROBE (Strengthening the Reporting of Observational studies in Epidemiology) statement [15]. Inclusion criteria were incisional hernia repair in our institution between September 2004 and September 2015 and age above 18 years. Exclusion criteria were loss to follow-up, missing written consent or different operation technique than IPOM repair.

Four patients were excluded because of mesh implantation in a sublay position. All patients eligible for inclusion were invited for clinical assessment in our outpatient department. If patients neither responded to phone calls nor to written convocation, contact was sought through the corresponding family doctor. Those patients not being able to attend clinical examination were interviewed by a standardized telephone questionnaire. All patients were examined and interviewed by the same independent investigator (SEB).

The primary outcome parameter was the incidence of hernia recurrence. Hernia recurrence was defined as proposed by Korenkov et al. [16]: “Any abdominal wall gap with or without bulge in the area of postoperative scar perceptible or palpable by clinical examinations or imaging.” Ultrasonography was used if clinical examination was not unequivocal.

Secondary outcome variables were operation time, length of hospital stay, frequency of SSI as defined by the Centers of disease Control and Prevention (CDC) [17], complications as defined by Dindo et al. [18], reoperation, chronic pain, and localization of hernia relapse as defined by the European Hernia Society (EHS) [19]. Chronic pain was defined as pain of 4 or more out of 10 points on a visual analogue scale for 3 months or longer at the time of investigation according to the International Association for the Study of Pain [20].

Patient-related factors such as age, sex, body mass index (BMI), hernia size, length of hospital stay, and operation related factors such as mesh size and operation time were extracted from the medical records. The study design was approved by the cantonal ethics committee of Bern, Switzerland (KEK 152/15), and informed consent was obtained from all patients.

### Surgical technique

Since 2004, laparoscopic incisional hernia repair was gradually introduced. The laparoscopic technique was applied for all patients with the exclusion of patients requiring additionally an intraabdominal open procedure. All hernia repairs have been performed with implantation of a coated non-resorbable mesh (68% Parietene™ composite (polypropylene), 15% Parietex™ composite (polyester), 9% DynaMesh® (polyvinylidene fluoride), 8% other). In laparoscopic incisional hernia repair, meshes were placed using IPOM technique as described previously [21, 22]. In open incisional hernia repair meshes were similarly placed intraperitoneal in IPOM position. Mesh fixation was done with Prolene 2-0 non-resorbable sutures (42%), tacks (10%) or both (48%) according to the surgeon’s choice.

### Statistical analysis

A 2:1 propensity score matching of the laparoscopic and the open IPOM group was performed. Matching criteria were age, sex, and BMI. Normality of distribution was assessed using the Shapiro–Wilk test. Categorical data were compared using Fisher’s exact test and continuous data using Mann–Whitney *U* test for non-parametric distribution and two-sample *t* test for parametric distribution. Recurrence rates were shown as Kaplan–Meier curve and compared by Log rank testing. A significance level of < 0.05 was assumed to be statistical significant. Statistical analysis was performed using SPSS Statistics version 25 (IBM Corporation, Armonk, United States).

## Results

**Fig. 1:**
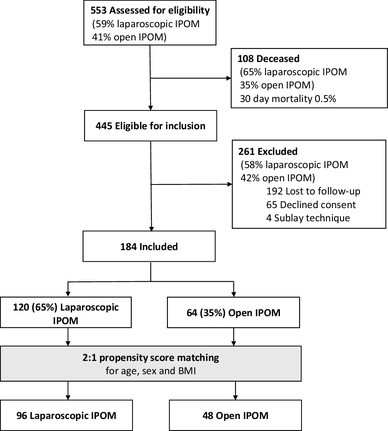
Flow chart of the recruitment process

Among 553 patients with incisional hernia, 326 (59%) underwent laparoscopic hernia repair and 227 (41%) underwent open hernia repair. A total of 184 patients were available for follow-up investigations and 120 (65%) patients underwent laparoscopic incisional hernia repair and 64 (35%) patients open incisional hernia repair. After propensity score matching, 96 patients remained in the laparoscopic IPOM group and 48 patients in the open IPOM group. A flow chart of the patient inclusion process is shown in Fig. 1. Patients who underwent conversion from laparoscopic to open surgery were analyzed on an as-treated basis. A total of 108 (75%) patients were assessed by clinical examination and 36 (25%) patients underwent a structured telephone interview. The baseline characteristics are shown in Table 1.

**Table 1:** Patient’s characteristics

	Laparoscopic IPOM (*n* = 96)	Open IPOM (*n* = 48)	*p* Value
Age, years, median (IQR)	65.5 (58.0–74.0)	67.5 (57.5–73.0)	0.920^a^
Sex, female/male, *n* (%)	31 (32)/65 (68)	17 (35)/31 (65)	0.712^b^
BMI, kg/m^2^, median (IQR)	27.2 (24.7–30.0)	27.5 (24.9–31.0)	0.812^a^
Hernia size, cm^2^, median (IQR)	25 (11–88)	29 (11–92)	0.735^c^
Primary incision			0.785^b^
Median, *n* (%)	67 (70)	31 (65)	
Transverse, *n* (%)	24 (25)	14 (29)	
Other, *n* (%)	5 (5)	3 (6)	

*IQR* interquartile range, *BMI* body mass index, *IPOM* intraperitoneal onlay mesh

^a^*t* test

^b^Fisher’s exact test

^c^Mann–Whitney *U* test

### Recurrence rates

**Fig. 2:**
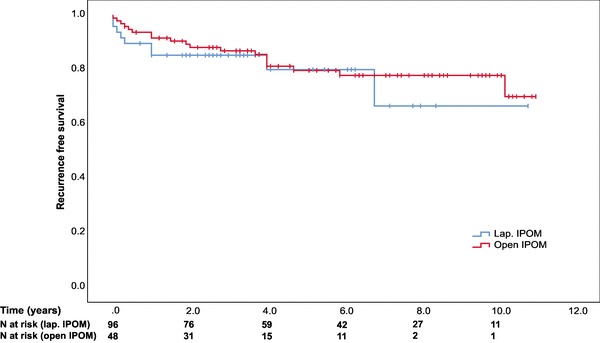
Kaplan–Meier curve of recurrence free survival stratified by operation technique (log rank *p* = 0.580)

**Fig. 3:**
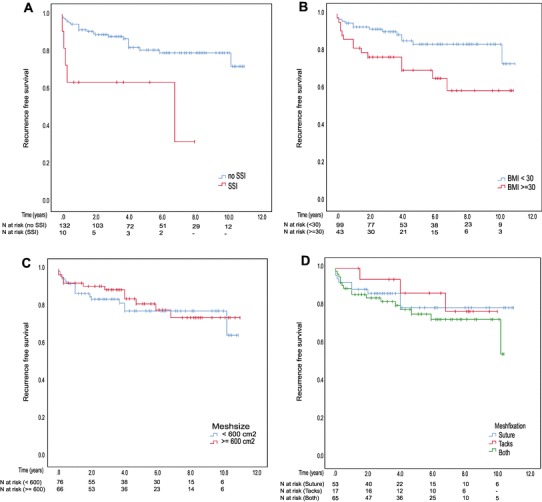
Kaplan–Meier curves of recurrence-free survival stratified by **A** surgical site infection (SSI) (log rank *p* = 0.002), **B** body mass index (BMI) (log rank *p* = 0.013), **C** mesh size (log rank *p* = 0.653), and **D** mesh fixation technique (log rank *p* = 0.586)

Overall recurrence rate was 20% (*n* = 19) in the laparoscopic group and 19% (*n* = 9) in the open group (*p* = 1.00) after a mean follow-up of 5.5 ± 3.0 years (Fig. 2). Recurrence rate was significantly increased in patients with SSI (log rank *p* = 0.002) and BMI ≥ 30 kg/m^2^ (log rank *p* = 0.013) but not dependent on mesh size or type of fixation (Fig. 3A–D).

### Secondary outcomes

Median operation time (120 vs. 180 min, *p* < 0.001) and median length of hospital stay (6 vs. 8 days, *p* = 0.002) were significantly shorter after laparoscopic incisional hernia repair. Complications (10 vs. 23%, *p* = 0.046) and SSI (1 vs. 21%, *p* < 0.001) were significantly fewer in laparoscopic compared to open repair (Table 2). There were no significant differences in mesh size, frequency of reoperation chronic pain, and overall complications between the two groups.

**Table 2:** Secondary outcomes

	Laparoscopic IPOM (*n* = 96)	Open IPOM (*n* = 48)	*p* Value
Operation time, min, median (IQR)	120 (100–180)	180 (136–265)	< **0.001**^**a**^
Length of hospital stay, d, median (IQR)	6 (4–7)	8 (5–12)	**0.002** ^**a**^
Mesh size, cm^2^, median (IQR)	500 (300–600)	525 (319–611)	0.332^a^
Reoperation, *n* (%)	6 (6)	2 (4)	0.620^b^
Chronic pain, *n* (%)	10 (10)	2 (4)	0.338^b^
Complication, *n* (%)	11 (10)	10 (23)	**0.046** ^**a**^
Grade I	1	–	
Grade II	1	1	
Grade III a	6	7	
Grade III b	2	2	
Grade IV a	–	1	
Grade IV b	–	–	
Grade V	–	–	
SSI, *n* (%)	1 (1)	10 (21)	< **0.001**^**a**^
Superficial	1	5	
Deep	–	4	
Organ space	–	1	

Bold values indicate statistically significant

*IQR* interquartile range, *SSI* surgical site infection, *IPOM* intraperitoneal onlay mesh

^a^Mann–Whitney *U* test

^b^Fisher’s exact test

### Localization of recurrence

**Fig. 4:**
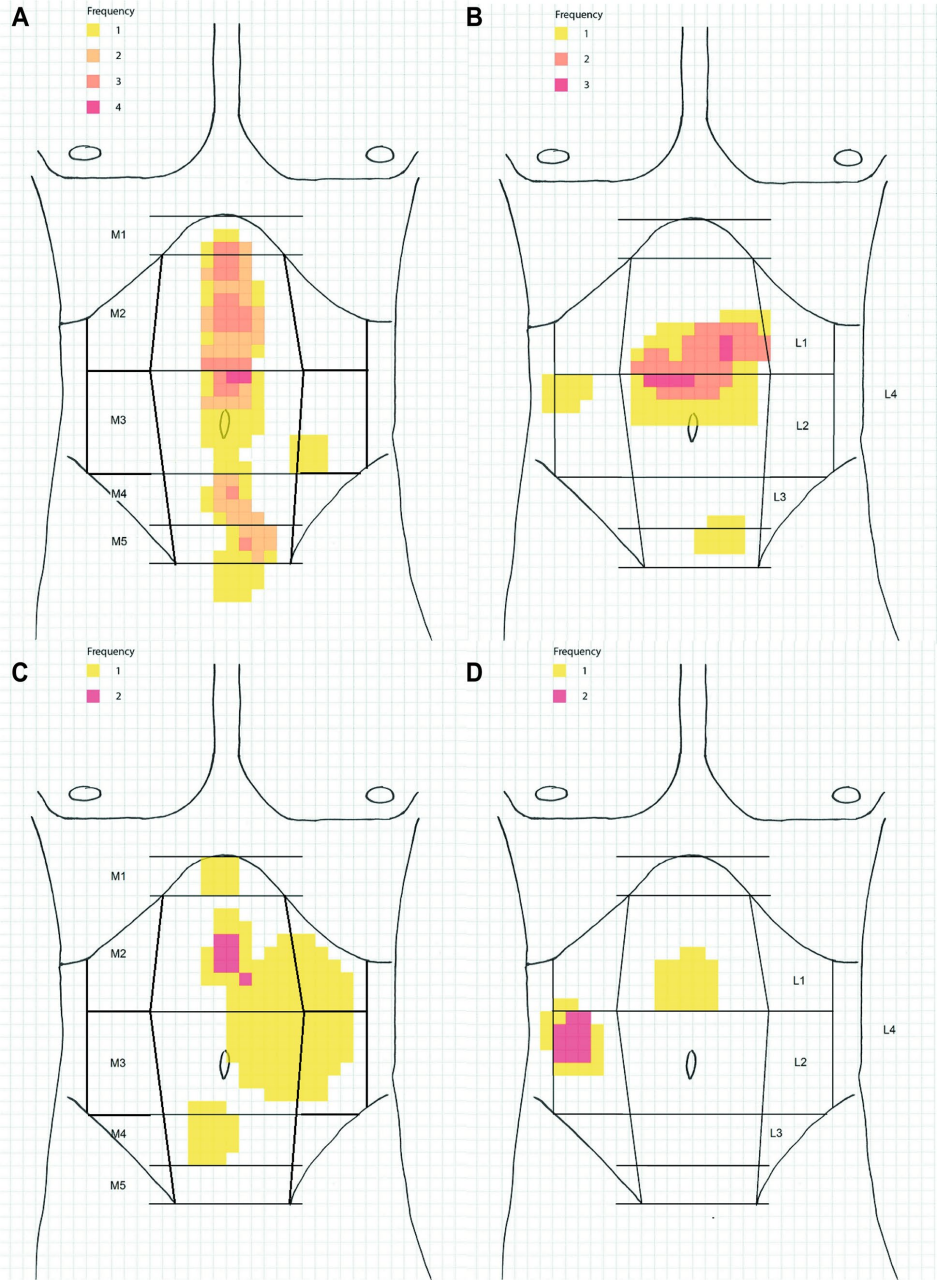
Frequency of the localization of incisional hernia recurrence after laparoscopic intraperitoneal onlay mesh (IPOM) repair in the median (**A**) and transverse (**B**) laparotomy and after open IPOM repair in the median (**C**) and transverse (**D**) laparotomy

Hernia recurrence in patients with median laparotomy as initial surgical access was mainly seen in the epigastric region (EHS classification M2) regardless if hernia repair was performed open or laparoscopically (Fig. 4A, C). In patients after oblique laparotomy as initial incision, recurrence after laparoscopic hernia repair was mainly seen in the epigastric region (EHS classification M2), whereas after open incisional hernia repair, hernia recurrence was mainly seen in the flank (EHS classification L2) (Fig. 4B, D).

## Discussion

To our knowledge, this is the first report that compares long-term results of laparoscopic versus open IPOM. The present study reveals the typical advantages of laparoscopic hernia repair: Shorter hospital stay and reduced SSI. Thus, avoiding mesh position as confounder, by including only IPOM in both arms, the present study supports the finding that primarily the access route provides these advantages [5–9, 11, 23].

### Recurrence rate

**Fig. 5:**
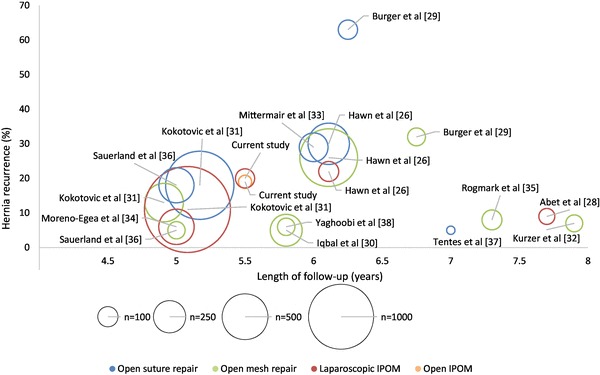
Incidence of hernia recurrence after incisional hernia repair in published studies with minimal follow-up 5 years since the year 2000

Recurrence rate was not significantly different between laparoscopic and open IPOM. Our long-term results extend the findings of meta-analyses with shorter follow-up ranging between 0.2 and 2.9 years that revealed no difference in recurrence rate [12, 24, 25]. Furthermore, we show that the recurrence rate after either laparoscopic or open repair reaches a steady state at 4.7 years postoperatively. An overview of studies reporting recurrence rates after incisional hernia repair after a follow-up of at least 5 years is given in Fig. 5 to set our findings in the context to the existing literature [26–37].

### Localization of hernia recurrence

Hernia recurrence after laparoscopic surgery was mainly observed in the epigastric region (EHS classification M2) of the median laparotomy scar. This might be because of difficulties to fix the mesh at the sternum and the ventral costal arch. Specifically after open incisional hernia repair, recurrence was additionally present in the flank (EHS classification L2) of the transverse laparotomy scar. Insufficient overlap at the transition to the retroperitoneum might be the reason. With its topographical maps of hernia recurrence, the study extends knowledge from the few previous studies, which described 18–29% of recurrences being off-midline [26, 38].

### Limitations

One limitation with this type of study is its limited rate of clinical follow-up because patients either were deceased, could not be contacted, or were unwilling to undergo clinical investigation at the time of recruitment. Another limitation of this study is its retrospective design with lack of randomization. Therefore, we used a propensity score matching approach to avoid selection bias.

## Conclusion

This study eliminates the bias of mesh position for the comparison of laparoscopic versus open incisional hernia repair. Laparoscopic IPOM revealed shorter operation time, hospital stay, reduced complications, and SSI when compared to open IPOM. In long-term follow-up, hernia recurrence is common in both techniques and occurs until 5 years postoperatively, independent of the operation technique.

## References

[1] Mudge M, Hughes LE (1985). Incisional hernia: a 10 year prospective study of incidence and attitudes. Br J Surg.

[2] Cassar K, Munro A (2002). Surgical treatment of incisional hernia. Br J Surg.

[3] Itatsu K, Yokoyama Y, Sugawara G, Kubota H, Tojima Y, Kurumiya Y, Kono H, Yamamoto H, Ando M, Nagino M (2014). Incidence of and risk factors for incisional hernia after abdominal surgery. Br J Surg.

[4] Flum DR, Horvath K, Koepsell T (2003). Have outcomes of incisional hernia repair improved with time? A population-based analysis. Ann Surg.

[5] Goodney PP, Birkmeyer CM, Birkmeyer JD (2002). Short-term outcomes of laparoscopic and open ventral hernia repair: a meta-analysis. Arch Surg.

[6] Asencio F, Aguilo J, Peiro S, Carbo J, Ferri R, Caro F, Ahmad M (2009). Open randomized clinical trial of laparoscopic versus open incisional hernia repair. Surg Endosc.

[7] Itani KM, Hur K, Kim LT, Anthony T, Berger DH, Reda D, Neumayer L, Veterans Affairs Ventral Incisional Hernia I (2010). Comparison of laparoscopic and open repair with mesh for the treatment of ventral incisional hernia: a randomized trial. Arch Surg.

[8] Misra MC, Bansal VK, Kulkarni MP, Pawar DK (2006). Comparison of laparoscopic and open repair of incisional and primary ventral hernia: results of a prospective randomized study. Surg Endosc.

[9] Forbes SS, Eskicioglu C, McLeod RS, Okrainec A (2009). Meta-analysis of randomized controlled trials comparing open and laparoscopic ventral and incisional hernia repair with mesh. Br J Surg.

[10] Rogmark P, Petersson U, Bringman S, Eklund A, Ezra E, Sevonius D, Smedberg S, Osterberg J, Montgomery A (2013). Short-term outcomes for open and laparoscopic midline incisional hernia repair: a randomized multicenter controlled trial: the ProLOVE (prospective randomized trial on open versus laparoscopic operation of ventral eventrations) trial. Ann Surg.

[11] Olmi S, Scaini A, Cesana GC, Erba L, Croce E (2007). Laparoscopic versus open incisional hernia repair: an open randomized controlled study. Surg Endosc.

[12] Sauerland S, Walgenbach M, Habermalz B, Seiler CM, Miserez M (2011). Laparoscopic versus open surgical techniques for ventral or incisional hernia repair. Cochrane Database Syst Rev.

[13] Liang MK, Holihan JL, Itani K, Alawadi ZM, Gonzalez JR, Askenasy EP, Ballecer C, Chong HS, Goldblatt MI, Greenberg JA, Harvin JA, Keith JN, Martindale RG, Orenstein S, Richmond B, Roth JS, Szotek P, Towfigh S, Tsuda S, Vaziri K, Berger DH (2017). Ventral hernia management: expert consensus guided by systematic review. Ann Surg.

[14] Holihan JL, Nguyen DH, Nguyen MT, Mo J, Kao LS, Liang MK (2016). Mesh location in open ventral hernia repair: a systematic review and network meta-analysis. World J Surg.

[15] von Elm E, Altman DG, Egger M, Pocock SJ, Gotzsche PC, Vandenbroucke JP, Initiative S (2007). The strengthening the reporting of observational studies in epidemiology (STROBE) statement: guidelines for reporting observational studies. Lancet.

[16] Korenkov M, Paul A, Sauerland S, Neugebauer E, Arndt M, Chevrel JP, Corcione F, Fingerhut A, Flament JB, Kux M, Matzinger A, Myrvold HE, Rath AM, Simmermacher RK (2001). Classification and surgical treatment of incisional hernia. Results of an experts’ meeting. Langenbecks Arch Surg.

[17] Mangram AJ, Horan TC, Pearson ML, Silver LC, Jarvis WR (1999). Guideline for prevention of surgical site infection, 1999. Centers for Disease Control and Prevention (CDC) Hospital Infection Control Practices Advisory Committee. Am J Infect Control.

[18] Dindo D, Demartines N, Clavien PA (2004). Classification of surgical complications: a new proposal with evaluation in a cohort of 6336 patients and results of a survey. Ann Surg.

[19] Muysoms FE, Miserez M, Berrevoet F, Campanelli G, Champault GG, Chelala E, Dietz UA, Eker HH, El Nakadi I, Hauters P, Hidalgo Pascual M, Hoeferlin A, Klinge U, Montgomery A, Simmermacher RK, Simons MP, Smietanski M, Sommeling C, Tollens T, Vierendeels T, Kingsnorth A (2009). Classification of primary and incisional abdominal wall hernias. Hernia.

[20] Classification of chronic pain (1986). Descriptions of chronic pain syndromes and definitions of pain terms. Prepared by the International Association for the Study of Pain, Subcommittee on Taxonomy. Pain Suppl.

[21] Kurmann A, Beldi G, Vorburger SA, Seiler CA, Candinas D (2010). Laparoscopic incisional hernia repair is feasible and safe after liver transplantation. Surg Endosc.

[22] Kurmann A, Visth E, Candinas D, Beldi G (2011). Long-term follow-up of open and laparoscopic repair of large incisional hernias. World J Surg.

[23] Eker HH, Hansson BM, Buunen M, Janssen IM, Pierik RE, Hop WC, Bonjer HJ, Jeekel J, Lange JF (2013). Laparoscopic vs. open incisional hernia repair: a randomized clinical trial. JAMA Surg.

[24] Al Chalabi H, Larkin J, Mehigan B, McCormick P (2015). A systematic review of laparoscopic versus open abdominal incisional hernia repair, with meta-analysis of randomized controlled trials. Int J Surg.

[25] Awaiz A, Rahman F, Hossain MB, Yunus RM, Khan S, Memon B, Memon MA (2015). Meta-analysis and systematic review of laparoscopic versus open mesh repair for elective incisional hernia. Hernia.

[26] Hawn MT, Snyder CW, Graham LA, Gray SH, Finan KR, Vick CC (2010) Long-term follow-up of technical outcomes for incisional hernia repair. J Am Coll Surg 210(5):648–655, 655–647. 10.1016/j.jamcollsurg.2009.12.038 () 10.1016/j.jamcollsurg.2009.12.038 20421023

[27] Abet E, Duchalais E, Denimal F, de Kerviler B, Jean MH, Brau-Weber AG, Comy M, Groupe d’etude et de recherche en chirurgie coelioscopique de lO (2014) Laparoscopic incisional hernia repair: long term results. J Visc Surg 151 (2):103–106. 10.1016/j.jviscsurg.2014.01.012 10.1016/j.jviscsurg.2014.01.012 24582727

[28] Burger JW, Luijendijk RW, Hop WC, Halm JA, Verdaasdonk EG, Jeekel J (2004). Long-term follow-up of a randomized controlled trial of suture versus mesh repair of incisional hernia. Ann Surg.

[29] Iqbal CW, Pham TH, Joseph A, Mai J, Thompson GB, Sarr MG (2007). Long-term outcome of 254 complex incisional hernia repairs using the modified Rives-Stoppa technique. World J Surg.

[30] Kokotovic D, Bisgaard T, Helgstrand F (2016). Long-term recurrence and complications associated with elective incisional hernia repair. JAMA.

[31] Kurzer M, Kark A, Selouk S, Belsham P (2008). Open mesh repair of incisional hernia using a sublay technique: long-term follow-up. World J Surg.

[32] Mittermair RP, Klingler A, Wykypiel H, Gadenstatter M (2002). Vertical Mayo repair of midline incisional hernia: suggested guidelines for selection of patients. Eur J Surg.

[33] Moreno-Egea A, Carrillo-Alcaraz A, Aguayo-Albasini JL (2012). Is the outcome of laparoscopic incisional hernia repair affected by defect size? A prospective study. Am J Surg.

[34] Rogmark P, Smedberg S, Montgomery A (2017). Long-term follow-up of retromuscular incisional hernia repairs: recurrence and quality of life. World J Surg.

[35] Sauerland S, Schmedt CG, Lein S, Leibl BJ, Bittner R (2005). Primary incisional hernia repair with or without polypropylene mesh: a report on 384 patients with 5-year follow-up. Langenbecks Arch Surg.

[36] Tentes AA, Xanthoulis AI, Mirelis CG, Bougioukas IG, Tsalkidou EG, Bekiaridou KA, Korakianitis OS (2008). Nuttall technique: a method for subumbilical incisional hernia repair revised. Langenbecks Arch Surg.

[37] Yaghoobi Notash A, Yaghoobi Notash A, Seied Farshi J, Ahmadi Amoli H, Salimi J, Mamarabadi M (2007). Outcomes of the Rives-Stoppa technique in incisional hernia repair: ten years of experience. Hernia.

[38] Janssen H, Lange R, Erhard J, Malago M, Eigler FW, Broelsch CE (2002). Causative factors, surgical treatment and outcome of incisional hernia after liver transplantation. Br J Surg.

